# Uptake and Translocation of Heavy Metals in Maize Leaves Exposed to Atmospheric Fallout

**DOI:** 10.3390/plants14223418

**Published:** 2025-11-08

**Authors:** Qiqi Wang, Hao Qi, Zhong Zhuang, Siyu Huang, Qi Wang, Yanan Wan, Huafen Li

**Affiliations:** State Key Laboratory of Nutrient Use and Management, Beijing Key Laboratory of Farmland Soil Pollution Prevention and Remediation, College of Resources and Environmental Sciences, China Agricultural University, Beijing 100193, China; wangqiqi7777888@163.com (Q.W.); wangqi88@cau.edu.cn (Q.W.); wanyanan@cau.edu.cn (Y.W.)

**Keywords:** heavy metal, maize, fallout, uptake, translocation

## Abstract

Atmospheric deposition is considered a source of heavy metals in plants. However, research on the uptake pathways of atmospheric particulate matter by leaves and the subsequent translocation of heavy metals within plants remains limited. In this study, the foliar uptake and translocation of heavy metals in two maize cultivars (fresh corn and silage corn cultivars, called Baiyunuo909 and Qingzhu932, respectively) were investigated through foliar exposure using soil from a mining area to simulate dry deposition under controlled chamber conditions. The height and biomass of maize were inhibited after three and five exposures to fallout deposition, and this inhibitory effect became increasingly pronounced with prolonged exposure. Furthermore, the activities of catalase (CAT) and superoxide dismutase (SOD), along with the malondialdehyde (MDA) content, significantly decreased in both cultivars relative to the control. This decrease was more significant in fresh maize, with the reduction ranges being 94.3%, 42.1%, and 40.8%, respectively. Fallout exposure elevated the contents of cadmium, lead, arsenic and zinc in the leaves, stems, and sheaths of both cultivars, despite no significant increase in the roots. The bioconcentration factors of leaves for heavy metals ranged from 0.0002 to 0.0007, representing a 3.5–fold variation; however, the overall low values showed no significant differences. Scanning electron microscopy with energy-dispersive spectroscopy revealed the accumulation of particulate matter on the leaf surface, with a higher density around the cuticle and stomata. Additionally, the fresh corn cultivar demonstrated greater sensitivity to fallout than the silage corn cultivar. In summary, heavy metals present in atmospheric particulate matter can be absorbed by leaves and subsequently translocated to other plant tissues. This study provides a theoretical foundation for understanding the mechanisms of foliar heavy metal uptake in maize.

## 1. Introduction

The rapid development of modern industry is frequently accompanied by environmental pollution, resulting in the release of substantial amounts of hazardous materials into the atmosphere [1,2,3]. Given that most heavy metals are highly toxic and resistant to degradation in ecosystems, increasing attention has been directed toward the issue of atmospheric particulate matter (PM) contaminated with heavy metals [4,5,6]. Heavy metals present in PM can accumulate on soil and plant leaves, posing a significant risk to food safety and human health [7,8,9]. In particular, PM_2.5_ is considered the most hazardous due to its high toxicity and potential for deep respiratory penetration [10,11].

Pan et al. [12] monitored heavy metals deposition via atmospheric particulate matter in farmland across North China from 2016 to 2020. Their results revealed that the annual atmospheric deposition flux of Pb in Beijing’s farmland (12 mg/m^2^) remained significantly higher than that in Europe (1 mg/m^2^). Similarly, Liang et al. [13] reported that the pollution was caused by heavy metals deposition from the near-surface atmospheric mercury (Hg) content in and around the coal gangue hills of the Wuda Coal Mine and was approximately 15–30 times the background level. Additionally, topsoil Hg content in areas adjacent to the gangue piles was significantly elevated compared to those farther away. Furthermore, studies have shown that heavy metals deposited from the atmosphere tend to occur in more bioavailable forms in topsoil and can transfer into plants through the atmosphere–soil–root pathway [14,15].

The uptake and translocation of heavy metals by plant roots from soil have been extensively studied; however, foliar uptake represents a more direct pathway for the transport of heavy metals from environmental sources into edible tissues. This pathway poses significant and often underestimated risks to food safety. Moreover, previous studies have indicated that even when heavy metal contents in soils are low, plants may still exhibit toxicity under specific conditions [16,17,18]. Several studies have reported that atmospheric deposition is a major source of heavy metals in plants, particularly in regions adjacent to mining and smelting operations [19,20,21]. Li et al. [22] found that atmospheric particles contributed up to 85% of the heavy metals in the leaves of cole (*Brassica chinensis* L.) and even 90% in those of capsicum (*Capsicum frutescens* L. var. longum Bailey). After four weeks of exposure to atmospheric deposition near a lead (Pb) smelter, the Pb contents in the shoots of lettuce, parsley, and ryegrass reached 122.0, 298.7, and 700.1 mg/kg (fresh weight), respectively [23], far exceeding the threshold established by the European Commission Regulation (EC) No. 221/2002 (0.3 mg/kg). According to a statistical analysis of the contribution rates of different sources to heavy metal accumulation in the edible parts of crops [24], the proportions attributed to atmospheric deposition were 63–83% for arsenic (As), 15–45% for cadmium (Cd), 71–80% for chromium (Cr), 10–53% for copper (Cu), 32–91% for Pb, 35–74% for zinc (Zn), and 24–34% for Hg. Therefore, it can be concluded that foliar uptake is a significant and non-negligible pathway for heavy metal accumulation in plants. However, research on this pathway remains limited, and the underlying mechanisms are still not well understood.

Currently, research on foliar uptake primarily focuses on leafy vegetables. As a staple food crop in China, maize (*Zea mays* L.) plays a crucial role in the dietary structure of the population. Moreover, maize leaves possess a relatively large surface area, enabling them to retain greater amounts of atmospheric particulate matter. Thus, investigating the uptake and translocation of heavy metals in maize leaves is of considerable relevance. In this study, two maize cultivars were selected for a greenhouse simulation experiment designed to investigate the foliar uptake and translocation of heavy metals in maize plants exposed to heavy metal-contaminated particulate matter. Due to frequent mining activities, the content of heavy metals in the soil of the mining area and surrounding farmlands often exceeds the standard, and pollution occurs frequently [25]. Therefore, soil in the mining area was adopted as the particulate matter for the simulation experiment. Specifically, the study aimed to (1) examine the differential characteristics of foliar uptake and translocation among different heavy metals; (2) compare the responses and accumulation patterns of heavy metals in fresh maize and silage maize under particulate matter exposure; and (3) elucidate the underlying mechanisms of foliar heavy metal uptake in maize. The findings are expected to contribute to reducing heavy metal accumulation in maize and promoting the safe production of agricultural products.

## 2. Materials and Methods

### 2.1. Experimental Design

A pot experiment was conducted in a greenhouse under controlled environmental conditions. The greenhouse was maintained with a day/night temperature regime of 25 ± 4 °C and 20 ± 2 °C, respectively, and the relative humidity in the greenhouse was 60–70%. A 14-h photoperiod was provided daily, with a uniform light intensity ranging from 240 to 350 μmol/(m^2^·s).

Two maize cultivars, Baiyunuo909 (B909, a fresh corn cultivar) and Qingzhu932 (Q932, a silage corn cultivar), were utilized in this pot experiment. The seeds were sterilized using 10% H_2_O_2_ for 30 min and then soaked in saturated CaSO_4_ for 8 h. Maize seeds were sown in a commercial potting mix at a density of four seeds per pot (containing 1.2 kg of nutrient soil), with four replicate pots per treatment. The soil characteristics were determined as follows: pH 6.44, organic matter 169 g/kg, cation exchange capacity 13.6 cmol/kg, total nitrogen 4.47 g/kg, Olsen-P 69.7 mg/kg, and available potassium 143 mg/kg. The total Cd, Pb, As, and Zn contents in the nutrient soil were measured at 0.15 mg/kg, 14.9 mg/kg, 8.92 mg/kg, and 47.9 mg/kg, respectively. These values are below the limits stipulated in the Chinese Soil Environmental Quality Standards (GB 15618–2018) [26].

**Figure 1:**
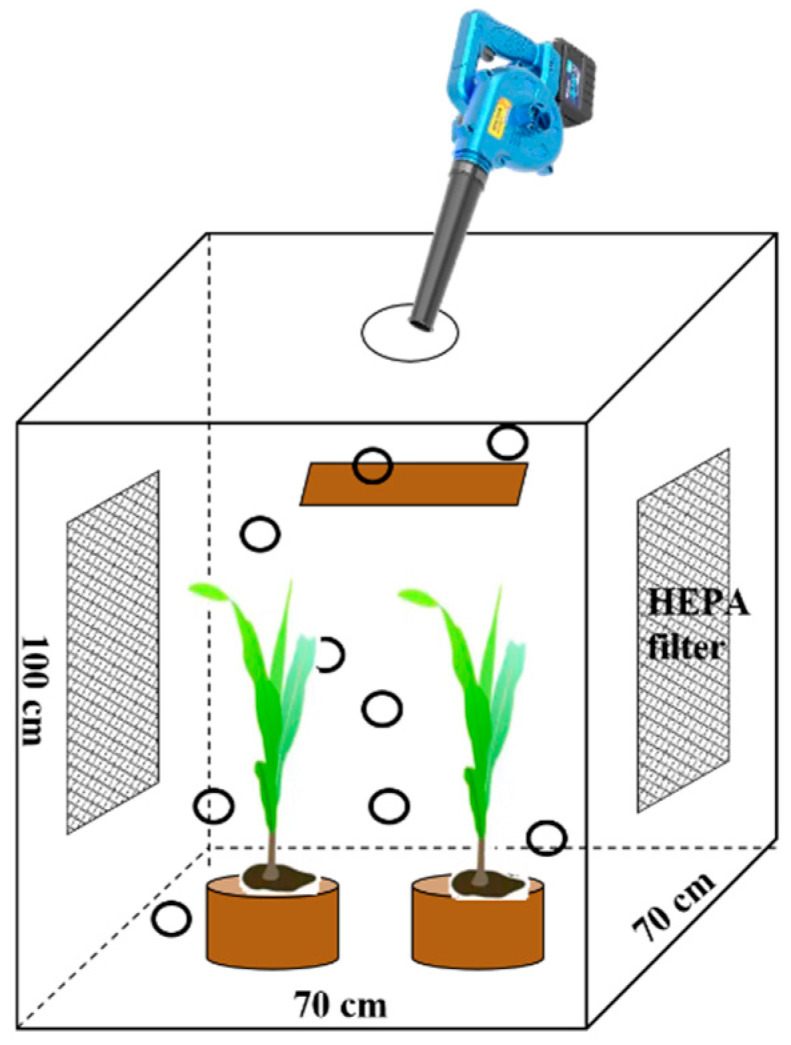
The graph of experimental equipment.

The exposure experiment was conducted in a transparent, sealed cuboid chamber measuring 70 cm in length, 70 cm in width, and 100 cm in height. High-efficiency particulate air (HEPA) filters were installed on two opposite sides of the chamber to filter particulate matter in the external atmosphere and ensure gas exchange, as illustrated in Figure 1. A blower was employed to disperse and diffuse the soil collected from a mining area into the chamber through a hole at the top of the chamber (peak air output 17.5 m^3^), simulating the process of atmospheric deposition. The soil was supported by a tray located 5 cm below the hole and was sieved through a 0.149 mm stainless steel screen to keep the heavy metal contents the same every time. Cd, Pb, As, and Zn contents in the soil used as particulate matter were 20.7 mg/kg, 867 mg/kg, 2651 mg/kg, and 4339 mg/kg, respectively.

Two treatments were established as follows: a control group where maize leaves were left untreated (CK), and a treatment group where maize was subjected to atmospheric particles smaller than 150 μm (T). The pots were covered with plastic lids with holes in which maize was placed and sponges filled the gaps to keep the soil free from particulate matter. The exposure regimen started after the third leaf had fully unfolded and was applied once per week. The air-blowing duration for each exposure was 5 min. Two maize plants were harvested following three and five exposure events, on the 30th and 45th day of growth, respectively.

### 2.2. Maize Growth Parameter

The fresh weights of the roots, stems, leaves, and sheaths of maize were measured following harvest. Plant height was also recorded on the 12th, 19th, 26th, 33rd, 40th, and 45th days after planting. These measurements were conducted to assess the effects of atmospheric deposition on maize growth.

### 2.3. Sample Collection and Heavy Metal Analysis

For the first collection, only the aboveground parts of the maize plants were collected and separated into stems and leaves. For the second collection, the plants were divided into four parts: roots, stems, sheaths, and leaves. The plant samples were washed thoroughly with deionized water, frozen in liquid nitrogen, and homogenized. A 0.4000 g aliquot of the sample was weighed into a microwave digestion tube. Subsequently, 8 mL of concentrated HNO_3_ was added, and digestion was carried out using a microwave digestion system (MARS5; CEM, Matthews, NC, USA). The total concentrations of Cd, Pb, and As in the digestion solutions were determined by inductively coupled plasma mass spectrometry (ICP-MS 7700; Agilent Technologies, Palo Alto, CA, USA). The Zn concentration was determined using inductively coupled plasma optical emission spectrometry (ICP-OES 8300; PerkinElmer, Waltham, MA, USA). Certified reference materials (GBW 10049) and blank samples were included for quality control. The recovery rates for Cd, Pb, As, and Zn ranged from 83 to 115%, 103–120%, 109–123%, and 85–113%, respectively.

### 2.4. Measurement of Antioxidant System Parameters

In total, 0.1000 g of maize leaves harvested on day 45 was weighed and homogenized mechanically in an ice-water bath with 0.9 mL of extraction solution (0.9% physiological saline or 0.1 mol/L phosphate buffer, pH 7.4). After homogenization, the mixture was centrifuged at 2500 rpm for 10 min, and the supernatant was collected for subsequent analysis. All steps were carried out at 4 °C.

The total protein (TP) content was determined using a commercial protein quantification assay kit (A045-2, Nanjing Jiancheng Bioengineering Institute, Nanjing, China). 0.05 mL of the supernatant was mixed with 3 mL of Coomassie Brilliant Blue working solution (prepared by diluting the stock solution with distilled water at a 1:4 ratio). After incubation for 10 min, the absorbance was measured at 595 nm using a microplate reader, with distilled water serving as the blank. The TP content was calculated and used for subsequent enzyme activity analyses. The results are expressed in g/L, representing the mass of total protein per liter of sample homogenate.

Catalase (CAT) activity was measured using a catalase assay kit (A007-1-1, Nanjing Jiancheng Bioengineering Institute, Nanjing, China). A 0.05 mL supernatant was mixed with 1 mL of reaction solution (containing 163 mmol/L H_2_O_2_ and 100 mmol/L (NH_4_)_6_Mo_7_O_24_) and incubated in a water bath at 37 °C for 1 min. The remaining H_2_O_2_ after the reaction formed a stable yellow complex with (NH_4_)_6_Mo_7_O_24_, and the absorbance was measured at 405 nm using a microplate reader. CAT activity was calculated based on the consumption of H_2_O_2_. The enzyme activity is expressed as U/mgprot, where one unit (U) is defined as the amount of enzyme that catalyzes the decomposition of 1 μmol of H_2_O_2_ per second per milligram of tissue protein.

Superoxide dismutase (SOD) activity was also measured using a catalase assay kit (A001-3, Nanjing Jiancheng Bioengineering Institute, Nanjing, China). A reaction system was prepared by mixing 0.02 mL of supernatant with 0.02 mL of enzyme working solution (prepared by mixing enzyme stock solution and enzyme dilution solution at a ratio of 1:10) and 0.2 mL of substrate reaction solution (prepared by mixing substrate stock solution and buffer solution at a ratio of 1:200). The mixture was incubated at 37 °C for 20 min, and the absorbance was measured at 450 nm using a microplate reader. SOD activity was calculated and expressed as U/mgprot, which represents the amount of enzyme required to achieve 50% inhibition of SOD in the reaction system per milligram of tissue protein sample.

The malondialdehyde (MDA) content was measured using a malondialdehyde assay kit (A003-1, Nanjing Jiancheng Bioengineering Institute, Nanjing, China). A 1 mL supernatant was mixed with 1 mL of a solution containing 0.5% thiobarbituric acid (TBA) and incubated in a 95 °C water bath for 40 min. MDA reacts with TBA to form a reddish-brown product, and the absorbance was measured at 532 nm using a microplate reader. The amount of MDA consumed was calculated and expressed in nmol per milligram of tissue protein (nmol/mgprot).

### 2.5. Scanning Electron Microscopy with Energy-Dispersive X-Ray Analysis

The morphology of the cuticle and stomata on the adaxial epidermis and the vascular bundle of leaf veins was observed using scanning electron microscopy (SEM) (Merlin Compact; Zeiss, Oberkochen, Baden-Württemberg, Germany) with energy-dispersive X-ray (EDX) (X-Max 20; Oxford Instruments, Abingdon-on-Thames, Oxfordshire, UK). The sixth maize leaves were collected and freeze-dried in a vacuum freeze dryer (FD-1A-50; Shanghai Zhengqiao Scientific Instruments Company, Shanghai, China) before SEM-EDX observation. After being placed on a copper substrate prepared with conductive adhesive and sputter-coated with gold, the plant samples were observed using SEM at voltages of 5 keV and 10 keV. Images were acquired at magnifications ranging from 500× to 5000×, with a resolution of 1280 × 960 pixels. The sizes of stomata and particles were calculated using ImageJ software 2.0.0. EDX analysis was performed on particle-covered areas to determine the atomic percentages of Na, S, Ca, Cu, Zn, As, Cd, and Pb.

### 2.6. Statistical Analysis

Based on the biomass and elemental content of both maize and particles, two parameters related to foliar uptake were calculated using the following equations (Equations (1) and (2)):

(1) Total amount = *B*_root_ × *C*_root_ + *B*_stem_ × *C*_stem_ + *B*_sheath_ × *C*_sheath_ + *B*_leaf_ × *C*_leaf_

(2) Bioconcentration factor of leaf (BCF) = (*C*_T-leaf_ − *C*_CK-leaf_)/*C*_PM_

where *B* is the biomass of the tissues; *C* is the heavy metal content in the tissues; *C*_PM_ is the heavy metal content in soil from the mining area.

All results are expressed on a fresh weight basis. Significant differences among treatments were determined using least significant difference (LSD) tests and Duncan’s ANOVA test at a significance level of *p* < 0.05. All statistical analyses were performed with SPSS 26.0, and figures were generated using R Studio 4.1.2.

## 3. Results

### 3.1. Growth of Maize and Antioxidant System Parameters

**Figure 2:**
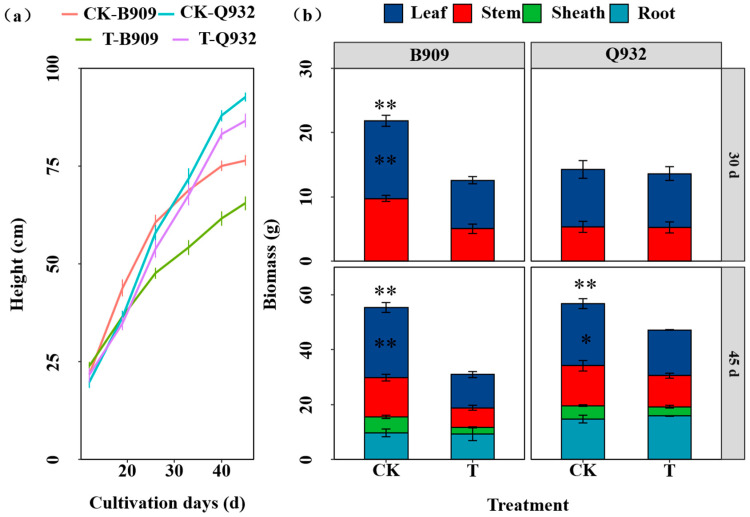
Effects of fallout exposure on the height (**a**) and biomass (**b**) of fresh maize (B909) and silage maize (Q932) after the first and second harvest. Data are presented as mean ± SE (n = 4). * and ** denote significant differences between treatments for the same cultivar within the same plant part and the whole plant (*p* < 0.05 and *p* < 0.01, respectively).

Maize growth was inhibited by exposure to fallout, and this effect became increasingly evident with prolonged exposure. Between the two cultivars tested, B909 demonstrated greater sensitivity to fallout than Q932 (Figure 2). The height of maize plants exposed to fallout was reduced by 14.4–21.5% in B909 and by 4.10–7.02% in Q932 compared to the control (Figure 2a).

Compared with the CK of B909, fallout exposure significantly decreased the total biomass of harvested maize both at the first and second time (Figure 2b) (*p* < 0.01). The total fresh biomass of maize B909 was reduced by 43.9% and 44.0% at the first and second harvest, respectively. Notably, the total biomass of Q932 showed no significant difference between treatments at the first harvest but had decreased by 17.0% at the second harvest (Figure 2b). Moreover, under control conditions, the biomass of the two cultivars differed significantly initially but became comparable by day 45. After 45 days of growth, the biomass of the four plant parts decreased in the following order: leaves > roots > stems > sheaths. The leaves accounted for 33.6–48.5% of the total biomass in B909 and 34.5–44.9% in Q932, respectively. The fresh weight of the leaves may have been the primary factor driving the change in total biomass, whereas the fresh weight of the roots remained unchanged compared to the control treatment.

**Figure 3:**
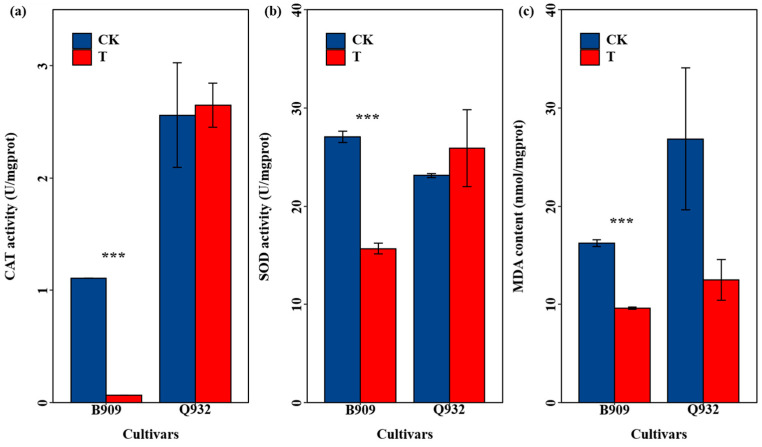
Activities of CAT (**a**), SOD (**b**), and content of MDA (**c**) of maize leaves. Data are presented as mean ± SE (n = 4). *** indicate statistically significant differences between the treatments for the same cultivar *p* < 0.001.

Furthermore, exposure adversely affected the antioxidant system in maize (Figure 3). The activities of CAT and SOD in B909 decreased significantly by 94.3% and 42.1%, respectively. Meanwhile, the MDA content decreased by 40.8%. For the silage maize Q932, there was no significant difference in the activities of CAT and SOD compared with the control treatment, while the MDA content decreased by 53.5%.

### 3.2. Contents of Heavy Metals in Maize

**Figure 4:**
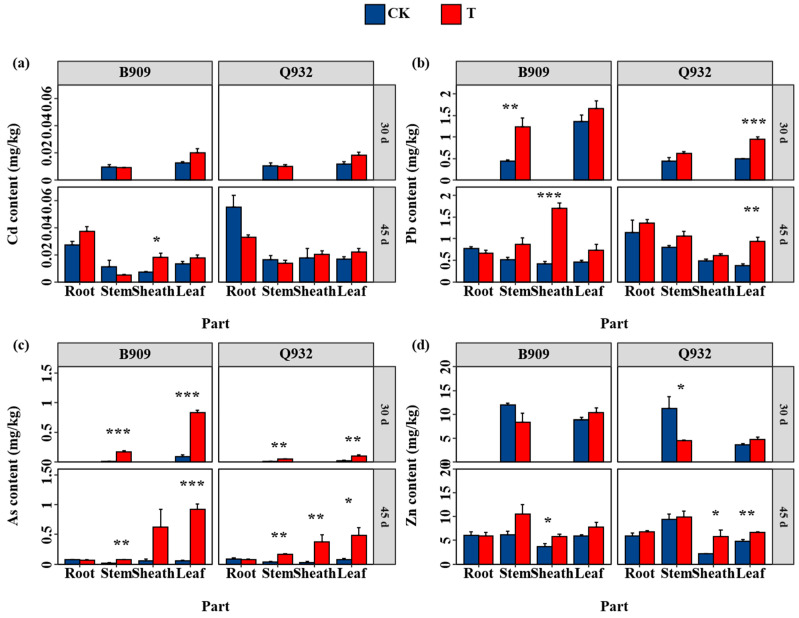
Contents of Cd (**a**), Pb (**b**), As (**c**), and Zn (**d**) in different parts of fresh maize (B909) and silage maize (Q932) after the first and second harvest. Data are presented as mean + SE (n = 4). *, **, and *** indicate statistically significant differences between the treatments for the same cultivar (*p* < 0.05, *p* < 0.01, and *p* < 0.001, respectively).

Maize exposed to fallout accumulated higher levels of heavy metals than the control; however, the accumulation trends varied among the different metals (Figure 4). The exposure to fallout resulted in an increase in Cd content in the leaves of both cultivars, representing a rise of 30.2% to 59.4% compared to the control. However, no significant differences were observed in the stems (Figure 4a).

Fallout exposure elevated Pb content in the leaves, stems, and sheaths of both cultivars, despite no significant increase in the roots (Figure 4b). At the first harvest (three exposures), the Pb content in the leaves and stems of B909 increased by 22.2% and 178%, respectively, compared to the control; corresponding increases for Q932 were 91.9% and 37.0%. Following the second harvest (after five exposures), the Pb contents in the aboveground parts of B909 and Q932 increased by 61.6–411% and 25.7–148%, respectively, relative to the control.

Fallout exposure led to extremely elevated As levels in the leaves, stems, and sheaths of both cultivars across two harvests (Figure 4c), although no significant increase was observed in the roots. On the 30th day of cultivation (following three exposures), the As content in the leaves and stems of B909 increased to 8.7-fold and 10.1-fold that of the control, respectively, while the corresponding increases in Q932 were 3.6–fold and 3.3–fold. After five exposures, the As content in the leaves, stems, and sheaths remained significantly higher than that of the control for both cultivars (*p* < 0.05).

At the first harvest, the Zn content in the leaves and stems showed no significant difference from the control. However, with prolonged exposure, the Zn content in the leaves, stems, and sheaths became significantly higher (Figure 4d). The Zn content in the leaves, stems, and sheaths of B909 increased by 31.9%, 98.1%, and 55.1%, respectively, while that of Q932 showed 38.3%, 5%, and 173% increase, respectively.

There was also a difference in the contents of heavy metals between the two cultivars after the second harvest; the contents of Cd and Pb in leaves of Q932 were 37% and 27% higher than those in B909. Conversely, after five exposure treatments, the leaf contents of As and Zn in B909 were 91% and 17% higher than those in Q932, respectively.

### 3.3. Distribution of Heavy Metals in Maize

**Figure 5:**
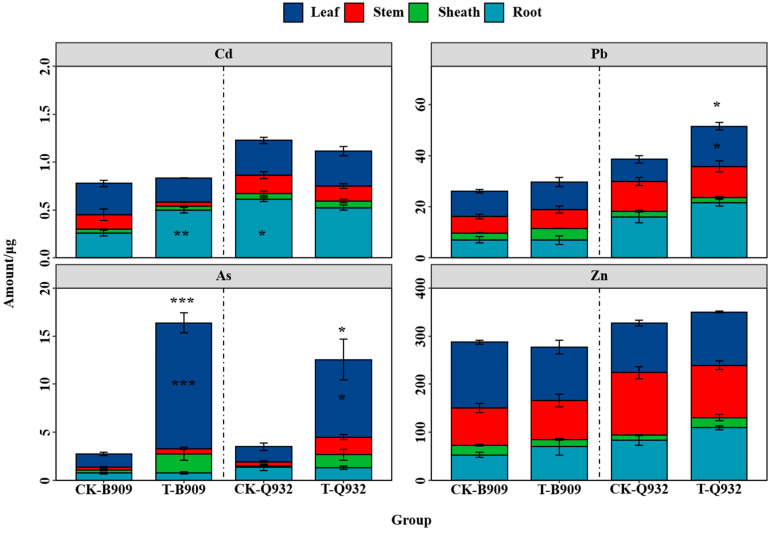
Amounts of heavy metals accumulated in fresh maize (B909) and silage maize (Q932) after the second harvest. Data are presented as mean ± SE (n = 4). *, **, and *** denote significant differences between treatments for the same cultivar within the same plant part and the whole plant (*p* < 0.05, *p* < 0.01, and *p* < 0.001, respectively).

The amount and distribution of heavy metals in the tissues of 45-day-old maize plants varied significantly across cultivars and elements (Figure 5). Although the particulate matter treatment did not significantly influence the Cd amount in the whole plant, total Cd in B909 increased by 6.94% and decreased by 9.24% for Q932 compared to the control (Figure 5). Additionally, the Cd accumulation in various tissues followed the order: roots > leaves > stems > sheaths. Specifically, the roots of B909 and Q932 accounted for 23.9–63.6% and 44.9–54.4% of the total amount, respectively.

While the treatment with particulate matter increased the total amount of Pb in the whole maize plants, this increase was more pronounced in the Q932 cultivar (Figure 5). The contribution of leaf Pb accumulation to the total amount was higher in B909 than that in its roots, whereas the opposite results were observed for Q932. Compared to the control, the total Pb amount in the leaves increased by 11.2% and 80.1% in B909 and Q932, respectively. In the roots, total Pb increased by 35.2% in Q932, while no significant difference was observed in B909.

The total As amount in both leaves and the whole maize of the two cultivars was significantly higher than that of the control. In the leaves, total As increased by 8.52 times and 5.12 times in B909 and Q932, respectively. As accumulation in leaves accounted for 41.5–87.6% in B909 and 35.8–71.8% in Q932 of the total As in maize plants.

There were no significant differences in Zn accumulation in the same parts of maize after PM exposure. Total Zn in B909 decreased by 18.7% and increased by 8.10% for Q932 compared to the control.

### 3.4. Bioconcentration Abilities of Heavy Metals

The bioconcentration factors of heavy metals in leaves from particulate matter are presented in Table 1. Regardless of the element type, the BCF showed no significant differences between the two cultivars. The leaves of Q932 (silage maize) exhibited a greater accumulation capacity for Cd and Pb compared to those of B909 (fresh maize), whereas the opposite trend was observed for As. Owing to the higher content in particulate matter, all BCF values were below 0.001. The highest BCF values recorded were 0.0004 for Zn in B909 and 0.0007 for Pb in Q932.

### 3.5. Morphology of Maize Leaves

**Figure 6:**
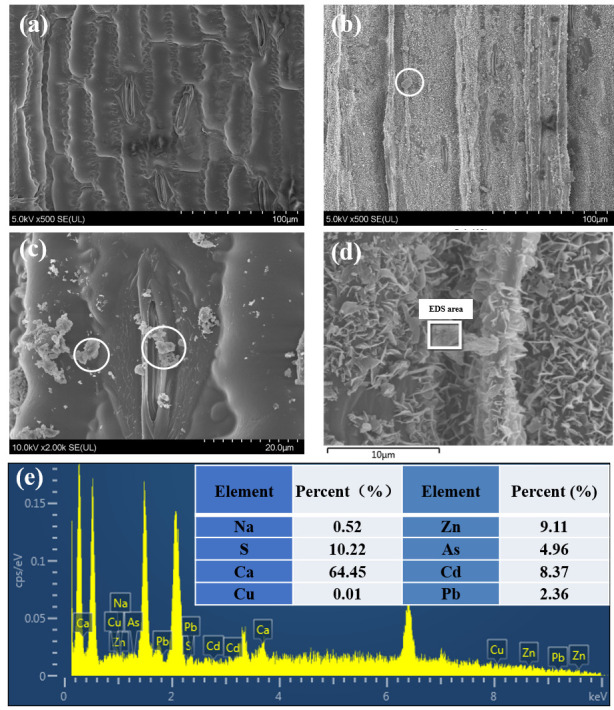
Scanning electron microscopy images of maize leaves. Imagery of the leaf adaxial epidermis in CK (**a**), T (**b**) under 500 magnifications, stomata in T under 2000 magnification (**c**), under 5000 magnification (**d**), and the corresponding EDS analysis (**e**). White circles indicate particulate matter, and the white area indicates the area where EDS analysis was conducted.

Under scanning electron microscopy, the morphology of the adaxial epidermis, including the cuticle and stomata, is presented in Figure 6. After exposure, the particulate matters were prominently observed on the surface and around the stomata, mostly in aggregated form. While the leaf surface of the control appeared clean with no attached particles, the distribution of stomata was relatively sparse (Figure 6a). At higher magnification (2000×), particles were observed deposited on and even within the stomata of exposed samples (Figure 6c). From the SEM image analysis, the average length and width of the stomata were measured to be 25.5 μm and 3.36 μm, respectively. Additionally, the size of the particles surrounding the stomata ranged from 0.56 to 5.85 μm. EDS analysis detected characteristic peaks of heavy metals, with atomic percentages of Cd, Pb, As, and Zn measured at 8.37%, 2.36%, 4.96%, and 9.11%, respectively (Figure 6e). This result confirms that the particulate matter located around or within the stomata originated from the simulated deposition treatment applied to the leaves in this experiment. Additionally, the particles were also abundant in Ca (64.45%) and S (10.22%).

## 4. Discussion

### 4.1. Pathway of Heavy Metal Uptake and Translocation in Leaves

Airborne heavy metals deposited on plant leaves can be released from particulate matter and subsequently absorbed by stomata and aqueous pores [27]. The foliar uptake of heavy metals by maize exposed to particulate matter was confirmed by the observed increase in their contents and total amount (Figure 4 and Figure 5). Exposure to fallout led to increased contents of Cd, Pb, As, and Zn in the maize leaves, reaching up to 30.2–33.4%, 61.6–148%, 554–1580%, and 31.9–38.3% above control levels, respectively (Figure 4). Similarly, after three weeks of exposure to particulate matter collected from a secondary lead smelter furnace, Cd, Zn, and Pb contents in cabbage shoots were 197, 4.64, and 61.2 times higher than those in the controls [28]. In a related study, Zhu et al. [29] calculated the Cd contribution to grains of rice in Xiangtan soils by comparing ^112^Cd/^111^Cd against the ^114^Cd/^111^Cd ratio; the average contribution was 63.55% of the atmosphere and 36.45% of the soil. In fact, studies have shown that the contribution of atmospheric deposition to heavy metals in plants depends on the metal content in both atmospheric particulate matter and soil.

In addition to the differences in heavy metal accumulation between the treatment and control, this study also used SEM to characterize the presence of particulate matter on the surface of maize leaves (Figure 6). The leaf surface was observed to be uneven, exhibiting numerous grooves that facilitate the deposition and adhesion of particles. It is well established that the initial stage of foliar uptake involves the physical entrapment of heavy metal–laden particles by the leaf surface structure [30,31]. This mechanism can serve as an effective means to capture airborne particulate matter and filter heavy metals from the atmosphere [32,33]. The trapping of particles by *Plantago lanceolata* was also found to be enhanced by the grooves present on the leaf surface [34]. Based on observations of the leaf morphology of eight common garden plants in the Hangzhou area, Shao et al. [35] concluded that leaves retain particles through the synergistic action of various microscopic structures, such as grooves, folds, chambers, and waxes. Furthermore, they noted that rougher leaf surfaces with more irregular folds and grooves exhibit a stronger capacity for particle retention.

After deposition on leaf surfaces, heavy metals from particles can enter the leaf via stomata, the cuticle, ectodesmata, and water stomata [36,37,38,39,40]. The stomatal pathway is clearly illustrated in Figure 6, where particulate matter that is smaller than the stomatal width can readily adhere to and enter the stomata. Previous studies using SEM have also documented stomatal uptake of foliar-applied substances in vegetables, rice, and wheat [23,41,42,43]. The epidermal cells of the substomatal region are thinner than the outer cuticular layer, which generally facilitates particle penetration via the stomatal pathway [44]. Furthermore, Gao et al. [45] reported that Chinese cabbage varieties with a higher stomatal length-to-width ratio exhibited greater accumulation of PM_2.5_-bound Pb through the stomata, highlighting the role of stomatal morphology in influencing foliar uptake efficiency. In a subsequent study, Gao et al. [46] revealed that PM_2.5_-bound Pb enters the leaves of Chinese cabbage primarily through stomata and undergoes a series of transformations into Pb(OH)_2_, glutathione-Pb and PbC_2_O_4_.

For maize leaves lacking observable trichomes, another main pathway for foliar uptake is through the cuticle, as lipophilic compounds can penetrate directly into the leaf tissue [47,48]. As illustrated in Figure 6b–d, PM is densely distributed within the cuticle. However, the pore diameter of the cuticle has been estimated to be less than 2 nm, making it difficult for PM to enter the leaf directly through these openings. While heavy metals may also penetrate the leaf interior following the dissolution of PM, depending on humidity, temperature, and PM characteristics [49,50]. Birbaum et al. [51] demonstrated that cerium (Ce) adsorbed onto maize leaves could not be removed by washing and was independent of stomatal opening under both light and dark conditions, thus confirming the cuticular pathway. Similarly, Mo et al. [52] also reported that the high accumulation of PM in the wax layer of the cuticle was related to the high levels of PM accumulation across all particle sizes.

Distinct patterns of uptake and translocation for different metals (Figure 4 and Figure 5) can be attributed to their unique uptake/translocation characteristics and varying contents in the PM. The BCF for heavy metals ranges from 0.0002 to 0.0007, representing a 3.5–fold variation (Table 1); however, the overall lower values suggest no significant difference in BCF among different elements in maize leaves. Compared to Cd and Pb, As was absorbed and accumulated more significantly in maize plants. This difference is primarily due to the greater variation in the elemental composition of the particles (Cd: 20.7 mg/kg; Pb: 867 mg/kg; As: 2651 mg/kg). The significantly lower accumulation of Cd in the plants may be attributed to the high Zn content in the soil (4339 mg/kg) (Figure 4). Zn can compete with Cd during root uptake by the plants, and such competition may also occur during leaf absorption and translocation. However, further investigation is needed to clarify the underlying mechanisms. The translocation factor of heavy metals shows no clear pattern, indicating that multiple factors control the transfer of heavy metals from leaves to other parts, which requires further in-depth research.

### 4.2. Variations in Heavy Metal Accumulation Between Cultivars

Maize growth was inhibited by exposure to fallout, with the fresh maize cultivar B909 showing greater sensitivity compared to the silage maize cultivar Q932. This inhibitory effect was directly reflected in the reduction in leaf and stem biomass and plant height (Figure 2). Fresh maize breeding prioritizes enhanced flavor and nutritional quality, which may come at the expense of investing resources into stress resistance pathways. In contrast, silage maize breeding focuses strongly on high biomass yield and resilience under environmental stress [53,54]. This fundamental difference in breeding goals provides a plausible explanation for our results.

Additionally, exposure negatively affected the antioxidant system in maize, as indicated by a significant reduction in the activities of CAT and SOD, as well as a decrease in MDA content (Figure 3). Research indicated that Cd exposure induced oxidative stress, which suppressed SOD activity. Consequently, the plant’s capacity to scavenge reactive oxygen species (ROS) was diminished, leading to escalated oxidative damage [55]. The results of the study by Wang et al. [56] indicated that treatments with high levels of nanoplastics and Cd significantly reduced the activity of SOD in leaves, and the differences in SOD activity might also be associated with the deficiency of trace elements such as Zn and Fe. Glutathione (GSH), characterized by its γ-glutamyl-cysteinyl-glycine structure, is prevalent in cells and represents a major pool of non-protein reduced sulfur. Its role in catalyzing bioreductive processes is essential for enhancing cellular defense against the toxicity of heavy metals and other foreign compounds [57]. A reduction in leaf Cd content, coupled with an enhancement in SOD, APX, POD, and GR activities, was observed in Mexican cotton following the supplementation of 50 μM GSH [58]. However, the content of glutathione was not detected in this study.

The adverse effects on maize growth may be attributed to both the physical impact and the toxicity associated with high levels of heavy metals in the fallout. PM can clog the stomata and lead to a decrease in stomatal conductance [59], which subsequently inhibits the photosynthetic rate and impairs material synthesis in plants [60]. Hong et al. [61] reported that exposure to CuO particles resulted in a significant reduction in the photosynthetic rate, stomatal conductance, and transpiration rate of cucumber leaves. Besides the physical blockage of stomatal openings by the particles, the high heavy metal content carried by the particles may also directly influence photosynthetic parameters and plant growth [39,62]. The toxicity of heavy metals absorbed through leaves is not yet fully understood and exhibits complex variability.

The translocation of heavy metals from the atmosphere to plant leaves is governed by several factors, such as stomatal conductance, cuticular properties, weather conditions, and the chemical forms of the metals within the particles [63,64,65,66]. In this study, two maize cultivars exhibited distinct patterns of heavy metal accumulation in their leaves (Figure 3). These findings align with research on Pb absorption from PM_2.5_ in different varieties of Chinese cabbage, where the high Pb-accumulating (HPA) type displayed a significantly greater stomatal length-to-width ratio compared to the low Pb-accumulating (LPA) type, corresponding to higher Pb accumulation in the shoots [45] (Gao et al., 2021). Similarly, Tomašević et al. [67] noted interspecific variation in particulate matter deposition on leaves, which they linked to differences in epidermal features.

## 5. Conclusions

The study demonstrates that atmospheric fallout deposition adversely affects maize physiology, with inhibitory effects on plant height and biomass becoming more severe with increased exposure duration. These effects are accompanied by reduced activities of CAT and SOD and a decrease in MDA content, suggesting an alteration in the plant’s stress response. Microscopic analysis confirms the deposition of particulate matter, particularly near stomata and the cuticle. Foliar uptake of heavy metals derived from particulate matter resulted in their accumulation in aerial plant parts. Notably, heavy metals can be transported within the plant following foliar uptake due to higher contents in stems and sheaths than in the control. Comparative analysis indicates that fresh corn is more sensitive to fallout exposure than silage corn. Silage corn is recommended to be cultivated in mining areas to decrease the health risk to human beings. In conclusion, leaves represent a major pathway for the uptake and translocation of heavy metals from atmospheric particulate matter into maize tissues. However, the mechanisms and drivers controlling the release of heavy metals from particulate matter after foliar deposition remain unclear and warrant further investigation. Additionally, future research should prioritize quantifying the contribution of foliar-absorbed heavy metals to maize grains and pay attention to the long-term ecological impact.

## Figures and Tables

**Table 1:** Bioconcentration factors of heavy metals.

Element	BCF	
	B909	Q932
Cd	0.0003 ± 0.0000a	0.0004 ± 0.0001a
Pb	0.0003 ± 0.0001a	0.0007 ± 0.0001a
As	0.0003 ± 0.0000a	0.0002 ± 0.0000a
Zn	0.0004 ± 0.0002a	0.0004 ± 0.0000a

Note: Data are presented as mean ± SE (n = 4). Different lowercase and uppercase indicate significant differences between cultivars in BCF and TF, respectively (*p* < 0.05, least significant difference).

## Data Availability

The data that support the finding of this study are available from the corresponding author upon reasonable request.
